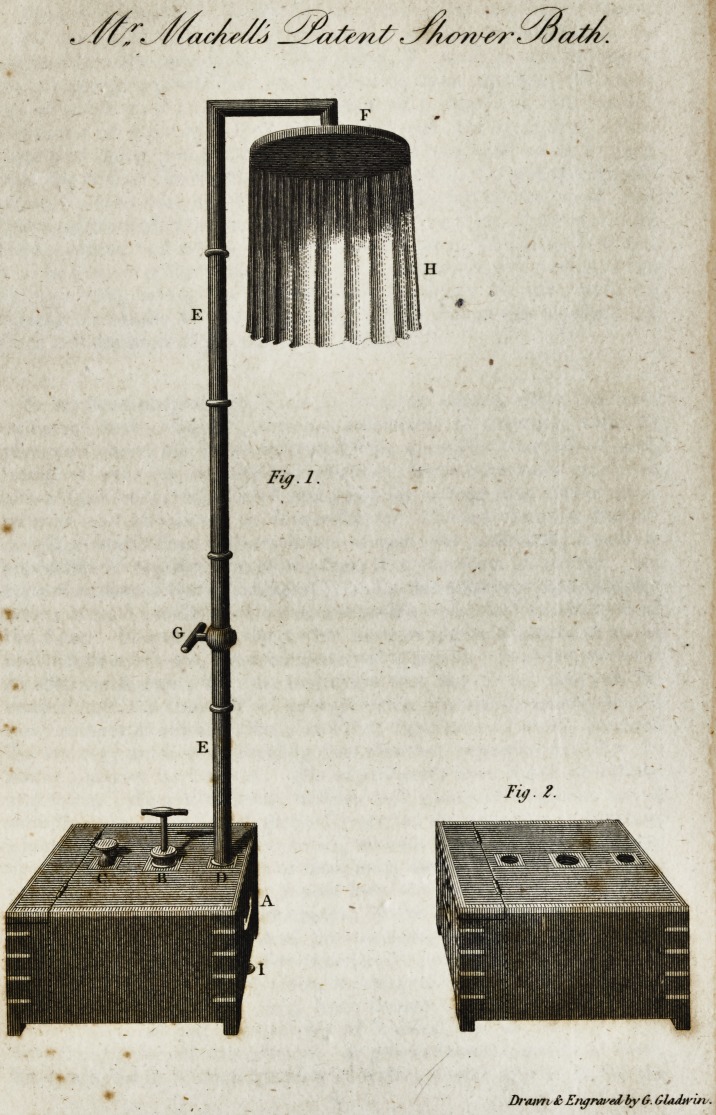# Medical and Physical Intelligence

**Published:** 1820-06

**Authors:** 


### Medical and Physical Intelligence.

TRANSACTIONS OF SCIENTIFIC SOCIETIES.

*ROYAL SOCIETY of London, March* 23.—A paper, by Mr. J. Hood, was read, entitled *on the Means of supplying Muscles in a State of Paralysis with Nervous Power.* The author having remarked the effects of nitrate of silver in removing the spasmodic action of the urethra, when applied to a stricture near its orifice, concluded that this salt has the property of influencing the action of the nerves at a considerable distance from the place where applied. Observing likewise the slight discharge produced by an eschar made by the nitrate of silver, he was induced to ascribe to it the power of exciting the absorbents to vigorous action by nervous communication, and in this manner he explained the good effects of the remedy in question in a case of diseased knee-joint, when applied so as to produce an eschar. Other cases were related, in which the external application of nitrate of silver proved stimulating to the nervous system, without proportionally increasing the action of the vascular system. Hence the author concluded, that muscular spasm and paralysis are caused by diminished nervous action; that muscular spasm cannot exist where the temperature is steadily above 90°; and that animal heat is produced principally by the action of the brain and nerves. Nitrate of silver, according to the author, applied to the head or spine, elevates the temperature, subdues spasm, and restores strength in certain paralytical cases; and, applied to enlarged joints, produces a more rapid absorption than any other remedy.

*April* 13.—A paper, by Sir E. Home, was read, *on the Milk-Teeth and Organs of Hearing of the Dugong.* The skull from which the following description was taken, and which is the only perfect one in Europe, was sent from Sumatra by Sir Stamford Raffles. The milk tusks of this animal resemble those of the narwhale and elephant, being, like them, deficient in external smoothness, when compared with the permanent tusks. But they are peculiar in having a shallow cup attached to their base, apparently for the purpose of receiving the point of the permanent tusks as soon as formed, and for directing them forward in the same course as that of the milk-tusks, and which is different from that in which the permanent tusks were originally directed. The milk-tusks of the dugong have hitherto been mistaken for its permanent tusks; but, as no full-grown individual has been yet examined, the form, &c. of the permanent tusks are unknown.

The grinding-teeth of this animal differ from those of all others. They consist of a double cone, the external crust of which is not enamel. This crust covers an internal harder coat, and the bulk of the tooth consists of soft ivory: hence, in wearing down, they will assume a concave form.

The organs of hearing also in this animal are quite peculiar. The malleus and incus are fastened to the sides of the tympanum by a bony substance extending across the intervening space. The stapes is opposed to, but not connected with, the foramen ovale, nor is it anchylosed with the ramus of the incus. The handle of the malleus projects in the centre of the circle over which the membranum tympani had been spread; and hence, in the recent animal, is probably attached to the centre of that membrane. As the habits of the dugong resemble those of the hippopotamus, Sir Everard was induced to examine the organs of hearing in the latter animal, to see if they were similar to those of the dugong. He found them, however, very different; the ossicula auditus being detached from the skull, and readily dropping out at the external orifice. In the dugong, the semicircular canals and cochlea are very small. Sir Everard was induced to conclude, from the above remarkable construction of the organs of hearing, that this animal, perhaps more than any other, hears by means of vibrations conveyed through the bones of the skull to the canals and cochlea.

*Royal Society of Edinburgh, Jan.* 3, 1820.—Sir GEORGE MACKENZIE, bart. read a paper, entitled *Speculations on the Nature of Sound.* The facts which he chiefly dwelt upon were, 1°, That, in every experiment, air has intervened between all bodies supposed to have the power of uniting and of conducting sound, and the apparatus of the ear; 2°, that sound varies in quality, and that the *quality* is not altered by transmission through different media, although the *intensity* of the sound may be increased or diminished; 3°, that the intensity of sound does not depend on the rate of vibration; 4°, that different substances have the power of modifying the quality of sound; 5°, that there are cases in which sounds interfere with one another; 6°, that the same effect, in acquiring any pitch of sound, may be obtained by preserving a spring of the same length, while the volume of air connected with it is altered in dimension, (as in playing on a Jew's harp;) or by keeping the volume of air the same, while the dimensions of the spring are altered, (as in the trumpet of an organ.) His opinions, as far as they were stated, are, 1°, That sound is a medium *sui generis;* 2°, that this medium is emitted by no other substance but air; 3°, that it is not conducted through air, but that successive portions of air, when put into a certain condition by impulse, emit it; and that, when the impulse reaches the air in connexion with the membrane of the tympanum, the sound emitted by that portion of air alone is made perceptible to us by the apparatus of the internal ear; and that we learn to judge of the distance of the body that gives the impulse, in a manner analogous to that by which we judge of the distance of objects in perspective; and that our acquaintance with sounds, as they proceed from impulse given by certain means, is derived from an imperceptible induction, similar to that by which a child learns the meaning of words, and forms correct ideas of distance. Lastly, that all means of producing sound are only means of causing air to emit it.

[We have thought it proper to introduce the above observations to our readers; but we must say that we are unable to derive any novel, or even clear, ideas from the opinions of the author. As well as we can understand his statements, he seems to consider sound in an abstract manner, as a body, instead of a mere mode of sensation from certain species of impulse on the auditory nerves, as physiologists must regard it.—EDIT.]

---

Dr. JOSEPH PAVON lately read to the *Academy of Sciences of Madrid*, an account of a new-discovered plant of South America, possessing qualities similar to those of cinchona. It is a shrub of a new genus, and has been called by Dr. P. *ananula febrifuga*, but it is known to the Indians by that of *chinininha.* It has been administered to several patients with intermittent fevers, by Drs. D. F. RUIZ, D. J. RUIZ, and DE LUZURIAGA, in doses of from a scruple to half a drachm of the powdered root every three hours. It is said to have removed cases which had resisted the cinchona.

---

WE insert a plate of an ingenious and very convenient apparatus for a shower-bath, which is the fruit of Mr. MACHELL's talents for mechanical invention. The following is a description of it, with which he has himself favoured us:

"Fig. 1, represents the patent shower-bath, as it appears when it is fitted together and ready for use.

"The body of this apparatus consists of a strong rectangular vessel, A, having a forcing syringe, B, in the middle of its top, and an orifice on each side of the syringe; one of which, C, is for the purpose of introducing the water or other fluid; and the other, D, has a series of pipes, E, sliding in and upon each other with screw-joints and leathers between them, so as, when screwed together, to form a water-tight tube, which conveys the water or other fluid to the canopy, F, by means of compressed air forced into the vessel by the action of the syringe. G is a stop-cock, for securing or discharging the water or other fluid; and the thumb-screw, I, will allow of any remaining portion of the fluid being drawn off after the operation of the shower; or, when necessary, the apparatus may be perfectly cleaned at this opening. H, is the oil-skin screen, being withdrawn on each side, in order to show the under or perforated side of the canopy, and which forms a drawer for the purpose of holding the screen, in packing-up the apparatus.

### Mr. Machell's Patent Shower Bath.

Fig. 1.

Fig. 2.

Drawn & Engraved by G. Gladwin.

Fig. 2, is a view of the apparatus in its most portable form, and which comprises the whole of the parts that are described in fig. 1.

*Method of using it.*—Take out the tubes at D, and invert them; screw three of them together, beginning at the largest, which must be screwed into the orifice, D. The canopy, F, must be screwed upon the two remaining small tubes after screwing them together; the lower end of which must be screwed, by means of a ring-joint, into the opposite end of the tube, which is connected with the apparatus. Previously to fixing this joint, the canopy must be placed so as to hang over the end of the vessel. Shut the stop-cock, G, and the thumb-screw, I; then take out the screw, C, on the top of the vessel, and introduce through its opening as much water or other fluid as will fill the apparatus, and return the screw into its place. The oil-skin screen is to be hung over the canopy; and, from sixty to eighty strokes of the syringe being made, the apparatus will be ready for use; and, by turning the handle of the stop-cock, G, the patient can regulate the force and duration of the shower at pleasure."

---

It has been observed by Taddei, that, on mixing either the deutoxide or deutochloride (corrosive sublimate) of mercury with the gluten of wheat, the latter separated from the water it contained, and, losing at once its viscosity and elasticity, became instantly converted into a hard body, capable of remaining for a long time under water without any dissolution or fermentation. He also observed that, on mingling wheat-flour with a solution of corrosive sublimate, it became impossible to separate the gluten from it in the form of strings. He concluded thence that the mineral retained this substance, and that, by means of it, it combined also with the starch and other constituents of the flour. It appeared, then, that flour or gluten might serve as an antidote to sublimate, when administered as poison. Some experiments he afterwards made, led him to believe that the gluten reduced the deutochloride to the state of protochloride (calomel), exactly in the same manner as albumen does; and that the gluten of wheat retained the mercury much more forcibly than albumen, recommended for the above purpose by Orfila. His experiments were made on rabbits and fowls. He administered, at first, two grains of sublimate mixed with flour or gluten, and gradually carried the quantity to fourteen grains in the course of ten hours, without causing the animals any apparent uneasiness. Some of them only, died at the end of three or more days, and he always found then the balls of gluten and the mineral entire in the digestive organs; which shows how easy it would be, after the administration of the antidote, to remove the whole by an emetic. Some comparative experiments proved that a single grain of sublimate, administered alone, would produce death in the above-mentioned animals; and that, from twenty to twenty-five grains of fresh gluten, or half the quantity of it dried, are necessary to neutralize one grain of the mineral. A proportionate quantity of flour, then, mingled in a small quantity of water, would appear to be the best antidote to the sublimate; and this can be more readily obtained than albumen in many situations. It would be prudent, indeed, for apothecaries to keep it ready in their shops for this purpose.

---

Mr. Barry, who has lately obtained a patent for his mode of evaporating vegetable extracts in a vacuum, has observed, during a comparison of the preparations made in this way and those commonly prepared, that phosphoric acid in a soluble state, is to be found in all the extracts. On further extending the investigation, it was ascertained that this acid, besides that portion of it which exists as phosphate of lime, is contained in a vast variety of vegetables; and he has also remarked, that all those vegetables which are cultivated seem to contain phosphoric acid in great abundance.

---

Dr. John, of Berlin, announces that he has obtained *succinic acid* by the following process:

Two pounds of bread, a pound and a half of honey, as much of the fruit of the ceratonia siliqua, two pints of vinegar, as much spirits, and twenty-eight pints of water, were treated in such a way as to obtain a liquid proper for undergoing the acetous fermentation. The vinegar produced was saturated with lime, and the acetate evaporated to dryness. Twenty-four ounces of this salt were triturated with an ounce of peroxide of manganese; the mixture was put into a retort, and subjected to distillation, after having been mixed with sixteen ounces of sulphuric acid diluted with thirteen ounces of water. When no more acid came over, the receiver was changed, and the fire augmented. A sublimate then condensed in the neck of the retort, which possessed the characters of succinic acid. When rectified, it crystallized in white flexible needles, and weighed two drachms.

John repeated this process two or three times, and always obtained succinic acid. The fruit of the ceratonia siliqua did not yield any succinic acid when subjected to analysis. Hence he is of opinion that the succinic acid obtained was formed during the process.

### REPORT OF DISEASES.

IT is difficult to say what have been the prevalent diseases during the last month. The applicants to the largest Dispensary in Westminster have been chiefly subjects of chronic diseases, especially of pulmonary affections. Most chronic diseases which terminate by inducing destructive irritation in the surrounding parts, as the whole class of tuberculous disorganizations, which constitute a great proportion of the maladies of the poor, assume an aggravated form at this season of the year. Patients in the last stage of phthisis proceed rapidly towards the common goal. The pure theorist thinks that blood-letting, refrigerants, rest, and abstinence from food, are mighty obstacles to their mortal progress; but the practitioner has learned to place but little reliance on them.

Another malady that has been apparently increased by the influence of the season, is epilepsy. Here abstraction of blood, especially by cupping on the nape of the neck, has afforded great relief in several cases, where there has been more pain in the head or alienation of mind than has usually attended the individual cases; and this practice has apparently favoured, considerably, the efficacy of other remedies.

Many children have rapidly sunk under what is called tabes mesenterica. We have not for a long time seen a case of this affection in which there were not signs of disease of either the mucous membrane of the intestines, or of the peritoneum, of a date apparently prior to that of the disease of the mesenteric glands. The disorganization of the mucous membrane of the small intestines has been very extensive in several cases, presenting ulcers of considerable size, some two or three inches in circumference, surrounded by a collection of caseiform matter, and great thickening of the mucous membrane; and it has been the mesenteric glands corresponding to such parts of the intestine that have been found enlarged and otherwise disorganized. These cases illustrate the principles that we have several times laid down in the late Numbers of this Journal, that inflammation of a mucous surface is followed by enlargement of those lymphatic glands whose vessels open upon it; a principle of the utmost practical importance, as it relates to the origin of phthisis from chronic pulmonary catarrh; and to tabes mesenterica from diarrhœa in children, when of long continuance, and when the irritation of the mucous membrane of the intestines on which it depends is aggravated and maintained by stimulating medicines or an irritating diet.

### METEOROLOGICAL JOURNAL.

**By Messrs. William Harris and Co. 50, Holborn, London.**

***From April 20 to May 19, inclusive.***

| Day of Month. | Moon. | Rain gauge | THERM. | | | BAROM. | | De Luc's HYGROM. Dry | Damp | WIND. | | ATMOSPHERIC VARIATION. | | |
|---|---|---|---|---|---|---|---|---|---|---|---|---|---|---|
| Apr. | | | | | | | | | | | | | | |
| 20 | ☽ | | 58 | 62 | 48 | 30·15 | 30·22 | 10 | 10 | NNW | NNE | Fine | | |
| 21 | | | 53 | 62 | 44 | 30·27 | 30·30 | 9 | 11 | NNE | ESE | Fine | | |
| 22 | | | 50 | 58 | 45 | 30·34 | 30·35 | 8 | 10 | ESE | ESE | Fine | | |
| 23 | | | 54 | 62 | 44 | 30·45 | 30·49 | 9 | 8 | ENE | ENE | Fine | | |
| 24 | | | 53 | 62 | 45 | 30·49 | 30·45 | 8 | 7 | NE | ENE | Fine | | |
| 25 | | | 50 | 61 | 42 | 30·40 | 30·27 | 6 | 7 | ENE | NE | Fine | | |
| 26 | | | 47 | 56 | 42 | 30·00 | 29·72 | 7 | 8 | WNW | NW | Fine | | Cloud. |
| 27 | | 100 | 44 | 45 | 39 | 29·72 | 29·92 | 10 | 8 | NE | N | Rain | Cloud. | |
| 28 | ○ | | 41 | 45 | 41 | 30·02 | 30·07 | 6 | 7 | NNE | WSW | Cloud. | Fine | Cloud. |
| 29 | | | 47 | 54 | 46 | 30·11 | 30·11 | 7 | 6 | WNW | WSW | Cloud. | Fine | Cloud. |
| 30 | | ·20 | 52 | 57 | 42 | 30·20 | 30·25 | 8 | 5 | NW | NE | Rain | Fine | |
| May | | | | | | | | | | | | | | |
| 1 | | | 46 | 52 | 45 | 30·31 | 30·30 | 7 | 8 | SSE | ENE | Foggy | Fine | |
| 2 | | | 52 | 57 | 37 | 30·27 | 30·15 | 7 | 8 | SW | ESE | Fine | Cloud. | |
| 3 | | | 43 | 51 | 45 | 30·11 | 30·08 | 9 | 8 | ESE | ESE | Cloud. | | |
| 4 | | | 50 | 54 | 38 | 29·98 | 29·87 | 7 | 8 | ESE | SE | Fine | Cloud. | |
| 5 | ☾ | | 46 | 50 | 40 | 29·85 | 29·90 | 7 | 9 | E | SE | Fine | Fine | Cloud. |
| 6 | | | 47 | 57 | 49 | 29·90 | 29·75 | 9 | 10 | W | SSW | Fine | Cloud. | Rain |
| 7 | | | 54 | 59 | 53 | 29·61 | 29·73 | 9 | 8 | NW | WSW | Cloud. | Fine | |
| 8 | | | 57 | 62 | 53 | 29·73 | 29·66 | 9 | 10 | WSW | S | Cloud. | Fine | Rain |
| 9 | | | 59 | 63 | 53 | 29·65 | 29·73 | 6 | 4 | SW | SSW | Fine | Cloud. | Fine |
| 10 | | | 62 | 65 | 53 | 29·80 | 29·90 | 1 | 2 | SW | SW | Fine | Cloud. | |
| 11 | | | 62 | 63 | 52 | 29·95 | 30·00 | 2 | 6 | SW | WSW | Fine | Cloud. | Fine |
| 12 | ● | | 59 | 65 | 49 | 30·04 | 30·02 | 6 | 8 | WSW | WSW | Cloud. | Cloud. | Fine |
| 13 | | | 59 | 64 | 59 | 29·98 | 29·90 | 8 | 7 | NE | SE | Cloud. | Fine | Cloud. |
| 14 | | | 66 | 67 | 51 | 29·86 | 29·85 | 7 | 6 | SSW | SSW | Cloud. | Fine | Cloud. |
| 15 | | | 58 | 65 | 48 | 29·84 | 29·80 | 6 | 7 | SW | SSW | Fine | Fine | Cloud. |
| 16 | | | 51 | 53 | 50 | 29·72 | 29·75 | 6 | 6 | WNW | SW | Rain | Sho'ry | Cloud. |
| 17 | | | 58 | 63 | 50 | 29·78 | 29·63 | 8 | 10 | WSW | SE | Cloud. | Fine | Sho'ry |
| 18 | | | 51 | 54 | 48 | 29·39 | 29·33 | 11 | 10 | SW | SW | Rain | | |
| 19 | | | 55 | 60 | 50 | 29·70 | 29·94 | 9 | 9 | W | SW | Fine | Rain | Cloud. |

**The quantity of rain fallen in April, is 2 inches and 83-100ths.**

## Figures and Tables

**Fig. 1. Fig. 2. f1:**